# Non-tuberculous mycobacterial infection and reactive dermatosis associated with adult-onset immunodeficiency due to anti–interferon-gamma autoantibodies: A case report

**DOI:** 10.1097/MD.0000000000021738

**Published:** 2020-09-04

**Authors:** Xiao-Na Liang, Yan-Fei Bin, Guan-Ting Lai, Ying-Hua Li, Jian-Quan Zhang, Xiao-Ning Zhong, Jing Bai, Mei-Hua Li, Jing-Min Deng, Zhi-Yi He

**Affiliations:** Department of Pulmonary and Critical Care Medicine, The First Affiliated Hospital of Guangxi Medical University, Guangxi Zhuang Autonomous Region, P.R. China.

**Keywords:** anti–interferon-gamma autoantibody, non-tuberculous mycobacterial infection, reactive dermatosis

## Abstract

**Introduction:**

Anti–interferon-gamma (anti–IFN-γ) autoantibody increases susceptibility to lower-virulence pathogens and causes immunodeficiency syndrome in HIV–negative patients.

**Patient concerns:**

A 69-year-old Chinese man presented with a 2-month history of pruritic skin lesions on his forearms, trunk, and legs. He was diagnosed with 5 opportunistic infections without conventional immunosuppression-associated factors in past. The most conspicuous characteristics were recurrent pulmonary infection, persistent immunoglobulin E elevation and eosinophilia during the whole disease course.

**Diagnosis:**

Enzyme-linked immunosorbent assay showed anti–IFN-γ autoantibody positive. The final diagnosis for the patient was adult-onset immunodeficiency due to anti–IFN-γ autoantibody, non-tuberculous mycobacterial (NTM) infection and reactive dermatosis.

**Interventions:**

The patient underwent long-term anti-NTM and corticosteroid maintenance treatment.

**Outcomes:**

The patient was followed for 2 years during which opportunistic infection no longer happened, the immunoglobulin E level and eosinophil count reduced, the autoantibody levels remained largely steady and lung lesions absorbed.

**Conclusion:**

Clinicians should be vigilant for NTM infection in patients with anti–IFN-γ autoantibodies, even when culture results are negative. Long-term anti–non-tuberculous mycobacteria and glucocorticoid regimens were effective.

## Abstract 2

Supplemental Digital Content is available in the text

## 1 Introduction

Adult-onset immunodeficiency associated with anti–interferon-gamma (anti–IFN-γ) autoantibodies, first reported in 2004^[1]^, is found predominantly in Southeast Asia. Patients with anti–IFN-γ autoantibodies are susceptible to lower-virulence pathogens, commonly non-tuberculous mycobacterial (NTM), *Salmonella, Burkholderia sp.*, *Talaromyces marneffei*, *Cryptococcus neoformans*, *Histoplasma capsulatum*, and varicella zoster virus^[2–5]^. The skin is affected most commonly, followed by the lymph nodes and blood^[6]^. We describe an anti–IFN-γ autoantibody–positive patient with NTM infection and intractable reactive dermatosis.

## 2 Case report

**Figure 1:**
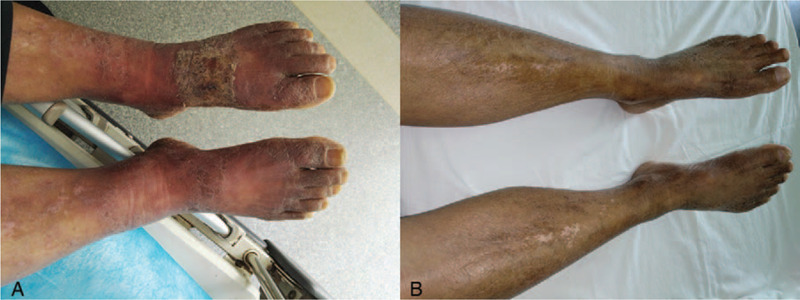
Skin lesions, erythema, and desquamation before (A; 4 November 2017) and after (B; 11 September 2018) glucocorticoid use, showing dramatic improvement and pigmentation.

A 69-year-old Chinese man presented with a 2-month history of pruritic skin lesions on his forearms, trunk, and legs in October 2017. The lesions showed thickening, erythema, and desquamation (Fig. 1A). From June 2015 to April 2017, he had intermittent fever and was diagnosed with five opportunistic infections without conventional immunosuppression-associated factors through culture or tissue biopsy (*Salmonella typhi*, *Mycobacterium* sp., *Candida* sp., *Burkholderia cepacia*, and *Talaromyces marneffei*; Table 1). The patient received chemotherapy for tuberculosis from October 2015 to October 2017, but the foci showed no absorption.

**Table 1:**
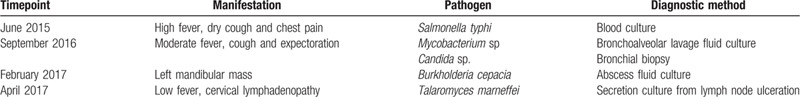
Clinical details for the 5 opportunistic infections.

**Figure 2:**
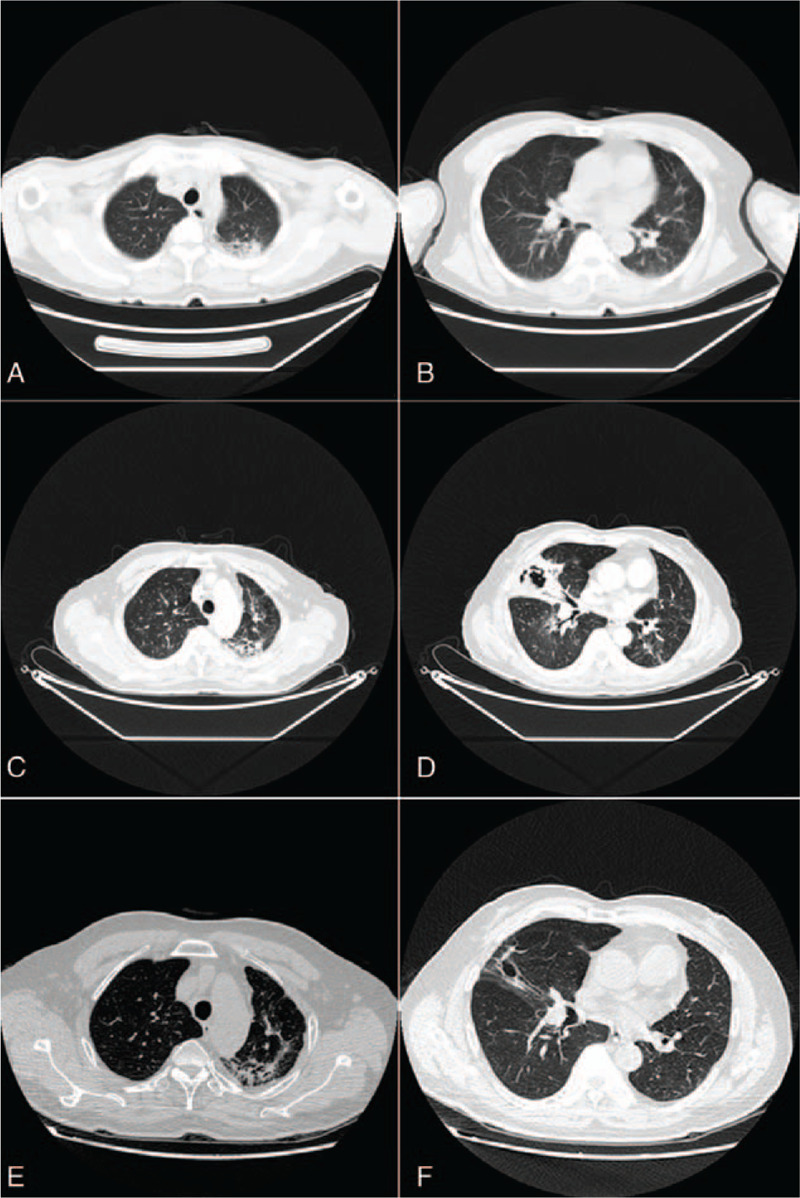
CT of the lungs revealed patchy shadows in the apical and posterior segments of the left upper lobe before anti-NTM treatment (A and B; 27 October 2017), new lesions in the lateral segments of right middle lobe after stopping anti-NTM treatment (C and D; 26 September 2018) and obvious absorption with regular anti-NTM and glucocorticoid use (E and F; 24 June 2019).

**Figure 3:**
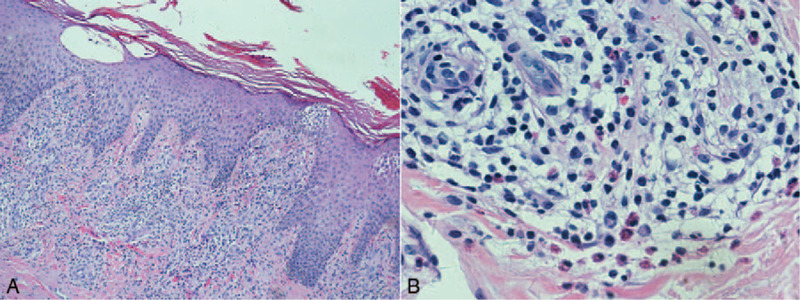
Histological examination of a skin biopsy sample showed nonspecific inflammation with lymphocyte infiltration and a few eosinophils in the edematous dermis (A, × 100; B, × 600; HE stain; 3 November 2017).

The patient's white blood cell count was normal (21.8% eosinophils; absolute eosinophil count, 1.70 × 10^9^/L). Immunological testing revealed elevated immunoglobulin (Ig)E (622.5 IU/mL) and IgG (30.2 g/L) levels, and reduced total T lymphocyte (49.70%) and CD8 cell (15.0%) concentrations. Ultrasound showed bilateral cervical, axillary, and inguinal lymphadenopathy. Chest computed tomography (CT) revealed patchy shadows in the apical and posterior left upper lobe, and enlarged mediastinal lymph nodes (Fig. 2A, B). Testing for parasites and human immunodeficiency virus (HIV) antibodies, and radionuclide bone imaging, yielded unremarkable findings. Bronchoscopic, lymph-node, and bone-marrow biopsies revealed no cancer or infection. Skin pathological examination showed nonspecific inflammation with lymphocyte infiltration and a few eosinophils in the edematous dermis, but no evidence of a pathogen (Fig. 3A, B). The patient was anti–IFN-γ autoantibody positive, as determined by enzyme-linked immunosorbent assay (see Text, Supplementary Method, which explains details for measuring anti–IFN-γ autoantibody levels).

The case was highly suspicious for NTM infection due to the patient's previous *Mycobacterium* positivity, the poor effects of anti-tuberculosis treatment, and the patient's anti–IFN-γ autoantibody positivity, which conferred a high susceptibility to NTM infection. Moxifloxacin (0.4 g/d) and clarithromycin (0.5 g/d) treatment was initiated, combined with antihistamines for eczematous dermatitis. The skin invasion had improved somewhat 10 days later. The patient continued the anti-NTM treatment for 2 months, and then elected to stop it. In the subsequent 3 months, he complained repeatedly of pruritis and erythematous edema, with persistent elevated IgE level and eosinophil count. Oral prednisone (30 mg/d) was initiated for reactive dermatitis due to anti–IFN-γ autoantibody positivity, and the skin symptoms improved dramatically (Fig. 1B). One month after initiation of prednisone treatment, the patient developed enlargement of the cervical lymph nodes and pain, and was restarted on anti-NTM treatment consisting of moxifloxacin and clarithromycin. Glucocorticoids were reduced gradually and stopped 8 weeks later, but the skin symptoms relapsed readily thereafter. Thus, corticosteroid treatment was resumed and adjusted according to these symptoms.

During 2 years of follow-up, the patient was hospitalized twice during glucocorticoid use for cough and worsening of lung lesions (visualized by CT, Fig. 2C, D). He had elected to discontinue the anti-NTM treatment before being admitted to hospital. To improve his skin symptoms and reduce the risk of infection, the anti-NTM and glucocorticoid regimens were maintained after discharge. Compound sulfamethoxazole and ethambutol were added, according to the patient's clinical condition. Thereafter, the patient's clinical condition remained stable, without opportunistic infection and with reduction of the IgE level and eosinophil count, largely steady autoantibody levels (Table 2) and lung lesions absorption (Fig. 2E, F). The Medical Ethics Committee of the First Affiliated Hospital of Guangxi Medical University approved this study [approval no. 2019 (KY-E-038)]. The Patient has provided informed consent for publication of the case.

**Table 2:**
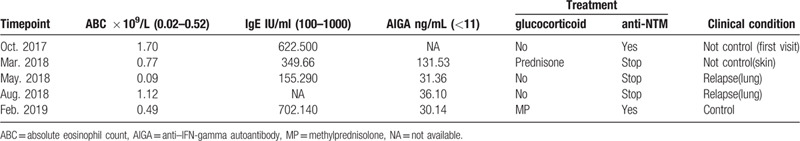
Summary of clinical parameters, treatment, and clinical conditions.

## 3 Discussion

Adult-onset immunodeficiency due to anti–IFN-γ autoantibodies is associated with opportunistic infections, commonly NTM infection and others^[3–5]^. Despite the lack of culture evidence, our patient's previous *Mycobacterium* positivity, susceptibility to NTM infection due to anti–IFN-γ autoantibody positivity, lung involvement, and therapeutic response support the diagnosis of NTM infection. Thus, we consider that this suspected diagnosis is credible. The patient required long-term anti-NTM treatment to prevent the development of lung lesions on progression. We highlights that clinicians should be vigilant for NTM infection in patients with anti–IFN-γ autoantibodies, even when culture results are negative.

Skin involvement, classified as reactive or infective dermatosis, is common in patients with anti–IFN-γ autoantibodies^[6]^. We considered our case to represent nonspecific reactive dermatosis, given the absence of histological evidence of infection. Persistent IgE elevation and eosinophilia were the patient's prominent characteristics. Absolute eosinophil counts were found to be significantly higher in patients with anti–IFN-γ autoantibodies with reactive skin lesions than in those with no reactive skin disease^[6]^. Given the skin biopsy evidence of eosinophil infiltration and the release of IgE-inducing histamine, proteases, and other mediators^[7]^, we suggest that the skin lesions were associated with IgE and eosinophil elevation. Some previous cases also showed remarkable elevated IgG^[5,8]^. Thus we guess that immunoglobulin elevation may be immune disorder associated with presence of anti–IFN-γ autoantibody. Reactive dermatosis treatment guidelines are lacking; corticosteroid was used in some cases to improve skin symptoms^[2,6]^. As our patient's skin symptoms relapsed readily after corticosteroid discontinuation, we consider that corticosteroids effectively suppress skin inflammation in patients with reactive dermatosis. However, more attention should be paid to balancing of the benefits of corticosteroid treatment and infection risk. Our patient underwent long-term anti-NTM and corticosteroid maintenance treatment, which improved his skin symptoms and minimized infection.

In summary, we reported the case of an HIV-negative man with anti–IFN-γ autoantibody who developed NTM infection and intractable reactive dermatosis. His dermatosis maybe relates to eosinophilia and elevated IgE. Long term anti-NTM and corticosteroid treatment improved clinical manifestations.

## Acknowledgments

*Medjaden* Bioscience Limited provided professional scientific editing service on this manuscript.

## Author contributions

Xiao-Na Liang, Ying-Hua Li, Xiao-Ning Zhong and Zhi-Yi He conceived and designed research; Yan-Fei Bin, Guan-Ting Lai, Ying-Hua Li, Jian-Quan Zhang, Mei-Hua Li and Jing-Min Deng collected data and conducted research; Xiao-Na Liang, Yan-Fei Bin, Guan-Ting Lai and Ying-Hua Li performed the experiment; Xiao-Na Liang, Guan-Ting Lai, Jian-Quan Zhang, Xiao-Ning Zhong, Mei-Hua Li, Jing Bai, Jing-Min Deng and Zhi-Yi He analyzed and interpreted data; Xiao-Na Liang and Yan-Fei Bin wrote the initial paper; Jian-Quan Zhang, Xiao-Ning Zhong, Jing Bai and Zhi-Yi He revised the paper.

## Supplementary Material

Supplemental Digital Content
