# Supplementary material for: Non-tuberculous mycobacterial infection and reactive dermatosis associated with adult-onset immunodeficiency due to anti–interferon-gamma autoantibodies: A case report
Source: Medicine (Baltimore). 2020 Sep 4;99(36):e21738. doi: 10.1097/MD.0000000000021738 (PMC7478425; doi:10.1097/MD.0000000000021738)
Supplement: Supplemental Digital Content [file medi-99-e21738-s001.docx]

**Supplementary method**

Serum samples from this patient and three self-reported healthy donors (two males, one female; age, 24–34 years) were collected and stored at –80°C for testing. Anti–IFN-γ autoantibody was determined by enzyme-linked immunosorbent assay (Cloud-Clone Corporation, Wuhan, China) according to the manufacturer’s instructions. The Medical Ethics Committee of the First Affiliated Hospital of Guangxi Medical University approved this study [approval no. 2019 (KY-E-038)].
